# Soil Reservoirs of Antifungal-Resistant Fungi: Implications for Plant Disease Management with a Focus on *Fusarium*

**DOI:** 10.3390/microorganisms14051018

**Published:** 2026-04-30

**Authors:** Ana B. Neves, Tiago M. Gonçalves, Artur Alves, Micael F. M. Gonçalves

**Affiliations:** Centre for Environmental and Marine Studies (CESAM), Department of Biology, University of Aveiro, 3810-193 Aveiro, Portugal; anab.neves@ua.pt (A.B.N.); tiago.martins.gon@ua.pt (T.M.G.); artur.alves@ua.pt (A.A.)

**Keywords:** *Fusarium*, antifungal resistance, resistance mechanisms, crop disease, IPM, resistance monitoring

## Abstract

Crop losses driven by fungal pathogens remain a major constraint to global food production, reinforcing agriculture’s dependence on fungicide-based disease control. Soil acts as a long-term reservoir and key hotspot for the evolution and persistence of antifungal-resistant *Fusarium*. The intensive, prolonged use of overlapping single-site fungicides in agriculture strongly selects for both intrinsic and acquired resistance in soilborne *Fusarium* populations, contributing to major crop losses, food insecurity, and One Health concerns. This review synthesizes current knowledge on (i) target-site (CYP51, β-tubulin, cytochrome b, SDH, myosin-5) and non-target-site (ABC/MFS efflux, multidrug resistance, epigenetic regulation) resistance mechanisms across the genus *Fusarium*; (ii) the influence of management practices and fungicide characteristics and behaviour in soil in reshaping microbial communities and selecting for resistant *Fusarium*; (iii) the consequences for plant disease management and the limitations of practices like cultural and biological control; and (iv) innovative strategies for plant disease management, as well as the monitoring and detection of antifungal resistance in soils. These aspects show that soil reservoirs of antifungal-resistant *Fusarium* are compromising fungicide-based control and increasing risks across sectors, highlighting the urgent need for sustainable, multi-layered, integrated pest management strategies combined with robust, molecularly informed resistance monitoring.

## 1. *Fusarium* as a Major Concern in Antifungal Resistance: Mechanism of Acquired and Intrinsic Resistance

*Fusarium* species are persistent soilborne fungal pathogens that pose a significant threat to various agricultural sectors, including cereals and ornamental plants [1,2,3], causing diverse and damaging symptoms such as vascular wilts, root and stem rots, cankers, and blights [4,5]. The genus *Fusarium* encompasses more than 300 phylogenetically distinct species, grouped into several species complexes (SCs), which creates a particularly challenging taxonomic landscape. Although these complexes exhibit varied geographic distributions, in some cases leading to endemic disease problems, such as coffee wilt disease in parts of Africa [6], the *F. solani* SC (FSSC), *F. oxysporum* SC (FOSC), and *F. fujikuroi* SC (FFSC) are ubiquitous. This global presence underpins major plant diseases worldwide, including Fusarium head blight (FHB) in cereals, Fusarium wilt in tomatoes, and sudden death syndrome in soybeans [7]. Several *Fusarium* species are also pathogens of woody hosts, significantly impacting fruit tree production and forestry. In forestry, *F. circinatum* is the causal agent of pine pitch canker, a severe disease affecting *Pinus* species globally. The infection causes resin-bleeding cankers, severe shoot dieback, and high seedling mortality [5]. In fruit tree pathology, *F. mangiferae* is the primary agent of mango malformation disease, which causes abnormal, sterile floral and vegetative shoot development, drastically reducing fruit yield and tree performance [8,9]. Similarly, the FSSC includes tree pathogens, such as *F. euwallaceae*, which affects both fruit trees, like avocado (*Persea americana*), and native forest trees. Vectored by the polyphagous shot hole borer (a species complex of ambrosia beetle), it causes Fusarium dieback, manifesting as vascular discoloration, branch wilting, and ultimately tree death [10].

**Figure 1:**
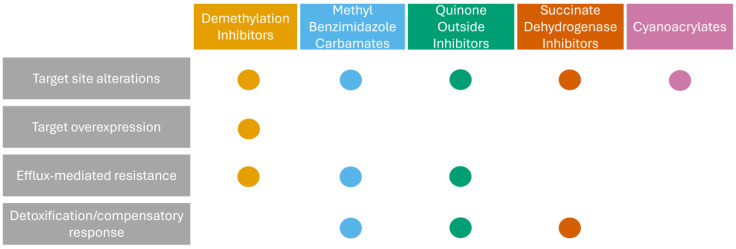
Overview of shared mechanisms of resistance to some of the main classes of fungicides.

Chemical control remains the primary line of defense against *Fusarium* diseases, although fungicide use patterns vary substantially by crop and region. Within the European Union (EU), for example, Northern countries tend to use higher volumes of imidazole and triazole fungicides, whereas Western and Southern regions rely more heavily on carbamates, dithiocarbamates, and inorganic fungicides [11]. These region-specific usage profiles exert different selective pressures on *Fusarium* populations, influencing the development of resistance. It is therefore essential to distinguish between intrinsic resistance, i.e., resistance naturally present at the species level without prior exposure to the fungicide, and acquired resistance, which arises in previously sensitive populations following stable, heritable selection events. Several *Fusarium* species exhibit significant intrinsic resistance to widely used fungicides, such as azoles, within the FSSC [12]. On the other hand, acquired resistance is increasingly documented, including widespread insensitivity to benzimidazoles and triazoles in *F. graminearum* [13,14] and resistance to prochloraz in *F. fujikuroi* [15]. These trends highlight the urgent need for systematic surveillance of fungicide susceptibility in field populations. While not an exhaustive review, this section highlights the most critical resistance mechanisms (summarized in Table 1 and Figure 1), acknowledging that our understanding remains limited and is complicated by the genus’s complex taxonomy, its remarkable genomic plasticity, and its complex stress response systems.

Currently, one of the most critical agronomic concerns involves Demethylation Inhibitors (DMIs or triazoles), such as tebuconazole, metconazole, and prothioconazole, which remain the backbone of FHB management in many regions [16,17]. Resistance to DMIs in *Fusarium* is multifactorial and closely linked to the unique architecture of the *CYP51* target gene. *Fusarium* species possess three paralogous genes: *CYP51A*, *CYP51B*, and the genus-specific *CYP51C*. Certain amino acid substitutions in these genes are thought to contribute to FSSC’s intrinsic resistance to many agricultural azoles [12]. Although the precise role of *CYP51C* has not been fully elucidated, its deletion increases sensitivity to specific inhibitors [18] and reduces virulence in *F. graminearum*, indicating a link between this paralog and fungal fitness [19]. In contrast to intrinsic mechanisms, acquired resistance in field populations of *F. graminearum* typically manifests as a quantitative shift in sensitivity rather than as fixed target-site mutations. This shift is commonly driven by inducible overexpression of *CYP51A*, *CYP51B*, or ATP-binding cassette (ABC) transporters (e.g., *FgABC3*). For instance, *F. pseudograminearum* and *F. graminearum* isolates resistant to prothioconazole and tebuconazole frequently exhibit strong upregulation of *CYP51A*, *CYP51B*, and transporter genes such as *ABC4*, without coding sequence mutations [20,21]. However, target-site mutations are well documented in other species. In *F. fujikuroi*, the S312T mutation in FfCYP51B is the primary mechanism conferring prochloraz resistance [15,22]. Some resistant isolates carry additional substitutions, such as the combined S312T and F511S mutations, which may impose fitness costs. However, the correlation between resistance, fitness, and pathogenicity remains inconsistent across studies and requires further investigation [23,24].

Resistance to Methyl Benzimidazole Carbamates (MBCs), including carbendazim, is mechanistically distinct and reflects the architecture of the *Fusarium* tubulin gene family, which includes two paralogs, *tub1* and *tub2*. Recent studies have clarified the differential binding targets of MBCs between these paralogs. Contrary to the paradigm in many other fungi, *β*_1_-tubulin, rather than *β*_2_-tubulin, is the preferred binding target of carbendazim in *F. graminearum* [25]. This target preference is driven by the fact that the *β*_2_-tubulin paralog naturally harbors a phenylalanine at position 240 (F240) in *F. graminearum*, which intrinsically lowers its binding affinity for MBCs compared to the leucine (L240) found in wild strains of most pathogenic fungi [26]. Because of these unique binding dynamics, mutations in either paralog can contribute to distinct resistance profiles. Intrinsic resistance in *F. verticillioides* has been linked to a Tyr50Asp mutation in *β*_1_-tubulin (the primary target) [27], whereas, conversely, acquired resistance in Asian clades is predominantly caused by point mutations in *tub2*. For instance, molecular characterization of *Fusarium* species causing sugarcane pokkah boeng disease demonstrated that field resistance to carbendazim is strongly associated with mutations in the *β*_2_-*tubulin* gene. Common examples of these target-site alterations include the F167Y mutation in *F. graminearum* [28] and E198T in *F. incarnatum* [29]. Additionally, transcriptional changes, detoxification enzymes, and drug efflux transporters contribute to the overall MBC resistance phenotype [30,31].

**Table 1:** Some major fungicide classes with respective modes of action, resistance status and mechanisms of intrinsic and acquired resistance in *Fusarium*.

Fungicide Class	Active Substance Examples	Mode of Action	*Fusarium* Resistance Status	Mechanisms of Resistance	References
Sterol Biosynthesis Inhibitors G1: Demethylation Inhibitors (DMIs)	Triazoles (difenoconazole, tebuconazole), triazolinthiones (prothioconazole), imidazoles (imazalil, oxpoconazole), pyrimidines, pyridines	Disruption of sterol biosynthesis in membranes (interaction with C14-demethylase (ERG11/CYP51))	Intrinsic resistance (e.g., *F. solani*)	Mutations in CYP51A, CYP51B and CYP51C protein sequences; potential roles of the unique *CYP51C* paralog.	[12,18]
			Acquired resistance (e.g., *F. graminearum*, *F. pseudograminearum*, *F. fujikuroi*)	Overexpression of *CYP51A*, *CYP51B*, and efflux pumps (e.g., *ABC3*, *ABC4*).	[20,21]
				Point mutations in CYP51B (e.g., S312T, F511S).	[15,22,23,24]
Benzimidazoles (MBC)	Carbendazim, thiophanate-methyl, benomyl, thiabendazole	Impaired functioning of cytoskeleton and motor proteins (inhibitor of β-tubulin assembly in mitosis)	Intrinsic resistance (e.g., *F. verticillioides*)	Tyr50Asp mutation in the *β*_1_-tubulin paralog.	[27]
			Acquired resistance (e.g., *F. graminearum*, *F. incarnatum*)	Point mutations in the *β*_2_-*tubulin* gene (e.g., F167Y, E198T).	[28,29]
Quinone outside Inhibitors (QoIs) (Strobilurins)	Azoxystrobin, kresoxim-methyl, pyraclostrobin)	Disruption of cellular respiration (targeting cytochrome bc1 at Qo site)	Intrinsic resistance (e.g., *F. graminearum*)	Rapid induction of alternative oxidase pathway (FgAOX).	[32]
			Acquired resistance (e.g., *F. fujikuroi*, *F. pseudograminearum*)	Point mutations in cytochrome b (*cytb*) (e.g., G143A, G143S, G137R).	[33,34,35]
Succinate Dehydrogenase Inhibitors (SDHIs)	Boscalid, penthiopyrad, fluopyram, benodanil, benzovindiflupyr, bixafen, pydiflumetofen, isoflucypram	Disruption of cellular respiration and ATP production (blocking succinate dehydrogenase)	Intrinsic resistance (*F. graminearum*)	Mediated by a paralog of *SdhC* (*FgSdhC1*) that guarantees enzyme function.	[36]
			Acquired resistance (*F. graminearum*, *F. asiaticum*, *F. oxysporum*)	Target-site mutations in subunits SdhA, SdhB, or SdhC (e.g., SdhA-Y182F, SdhB-H53Q).	[37,38,39]
Cyanoacrylates	Phenamacril	Inhibition of cell skeleton/motor proteins (targeting class I myosin)	Intrinsic resistance (*F. solani*, *F. oxysporum*)	Natural polymorphisms in myosin-5 (e.g., T218S, K376M, V151A).	[40,41]
			Acquired resistance (e.g., *F. graminearum*, *F. fujikuroi*)	Point mutations in the *myosin-5* gene.	[42,43]
			Multidrug Resistance (MDR) (Cross-resistance to, e.g., triazoles, SDHIs, QoIs, cyanoacrylates)	Overexpression of efflux pumps (ABC and MFS superfamilies) regulated by transcription factors (e.g., FgAtrR).	[44,45,46,47]

Some *Fusarium* species also exhibit high intrinsic resistance to Quinone outside Inhibitors (QoIs, or strobilurins). A major complication of QoI use is that these compounds can trigger the accumulation of the mycotoxin deoxynivalenol (DON) in *F. graminearum*, making them risky for FHB management [48]. Intrinsic resistance to azoxystrobin in *F. graminearum* has been attributed to rapid induction of the alternative oxidase pathway (FgAOX), which allows mitochondrial respiration to bypass inhibition of complex III [32], rather than to natural genetic mutations at the Qol target site. Indeed, the classic cytochrome b (*cytb*) resistance mutation, G143A, is notably absent in *F. graminearum* field isolates because a group I intron immediately follows codon 143. Because codon 143 is located at the 3′ end of the exon upstream of this self-splicing intron, it is needed for base-pairing with the intron to form the RNA secondary structure required for self-excision [49]. This means that a nucleotide substitution associated with the G143A mutation would disrupt this binding, preventing splicing out of the intron and resulting in a defective, lethal *cytb* transcript [50,51]. Conversely, other species lacking this constraint, such as *F. fujikuroi*, *F. pseudograminearum*, and *F. oxysporum* f. sp. *lycopersici*, develop acquired QoI resistance through mutations such as G143A, G143S, or G137R [33,34,35].

Management strategies based on Succinate Dehydrogenase Inhibitors (SDHIs) have evolved significantly in recent years. Some *Fusarium* species display intrinsic resistance to both earlier-generation SDHIs, such as boscalid, and novel broad-spectrum compounds, such as pydiflumetofen and fluopyram. For example, *F. graminearum* possesses a paralog of *SdhC* (*FgSdhC1*) that maintains succinate-ubiquinone oxidoreductase activity during SDHI exposure, providing a mechanism for intrinsic tolerance to some new generation SDHI compounds [36]. Despite this, the many novel broad-spectrum compounds show improved efficacy against major pathogens [52,53]. However, resistance is rapidly emerging under selective pressure, primarily driven by target-site mutations in the SdhB, SdhC, and SdhD subunits. For example, laboratory mutants of *F. graminearum* resistant to pydiflumetofen harbor various substitutions (e.g., SdhA-Y182F, SdhB-H53Q, SdhC-S31F), often associated with fitness costs [37]. In contrast, resistance in *F. asiaticum* and *F. oxysporum* does not always impose a fitness cost, suggesting a higher field risk of SDHI resistance for these species [38,39].

Phenamacril (JS399-19), a cyanoacrylate fungicide developed specifically for *Fusarium* management, offers a distinct mode of action by inhibiting myosin-5 ATPase activity. *F. solani* and *F. oxysporum* exhibit intrinsic resistance due to naturally occurring polymorphisms (e.g., T218S, K376M in *F. solani* and V151A and S418T in *F. oxysporum*) that prevent binding to the target site [40,41]. In contrast, while highly effective against naturally sensitive species, resistance has emerged in *F. fujikuroi*, *F. incarnatum*, and *F. graminearum* via point mutations in the *myosin-5* gene [42,43]. Importantly, phenamacril does not display cross-resistance with benzimidazoles or triazoles, supporting its use as a rotation partner in resistance management programs [54].

Beyond target-site alterations, *Fusarium* species adapt through complex regulatory networks. Epigenetic mechanisms, such as the chromatin-remodeling Swi/Snf complex, have been implicated in azole resistance in *F. graminearum* [55,56]. Although epigenetic regulation is expected to play a key role in fungal adaptation to stress, potentially promoting the persistence and spread of resistant strains, empirical evidence in *Fusarium* remains limited. Furthermore, Multidrug Resistance (MDR), defined as non-susceptibility to fungicides from at least two structurally different classes, is a growing concern. MDR phenotypes often result from the overexpression of membrane-associated efflux pumps, particularly those belonging to the ATP-binding cassette (ABC) and Major Facilitator Superfamily (MFS) superfamilies [57], which prevent intracellular drug accumulation. In *F. graminearum*, this mechanism facilitates cross-resistance to triazoles, SDHIs, cyanoacrylates, and QoIs [44]. Regulation of these transporters is mediated by transcription factors like FgAtrR, which acts as a dual regulator by simultaneously upregulating the azole target *FgCYP51A* and efflux pump genes, thereby promoting both specific (azole) and broad-spectrum (MDR) resistance [45]. Since these transporters also play critical roles in virulence and mycotoxin secretion, including DON [46,47], they appear to function as central components of the fungal stress response. This highlights the need to clarify how environmental pressures and agricultural practices shape the evolution of resistance in *Fusarium* populations.

A limitation in the current literature regarding *Fusarium* resistance mechanisms is the inconsistency of fitness cost evaluations associated with target-site mutations. Because of the genomic plasticity present in *Fusarium* species, a mutation that incurs a severe fitness penalty in one isolate may be easily compensated for in another. It would thus be important that future mechanistic studies move away from analyzing single isolates and instead adopt standardized, multi-strain fitness assays across diverse genetic backgrounds. Additionally, future research must prioritize transcriptomic studies that identify the specific environmental and agronomic triggers responsible for many aspects of MDR phenotypes.

## 2. Soil as a Reservoir of Antifungal-Resistant *Fusarium*: Microbiological Composition and Agricultural Drivers

**Figure 2:**
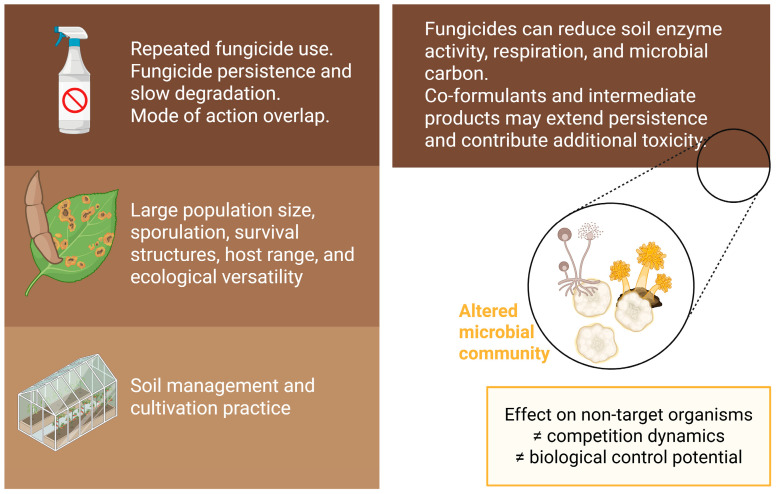
Key aspects driving antifungal resistance in soil and their connection to changes in soil microbial communities.

Fungicides have been shaping agriculture for the last 3000 years, evolving from elemental sulphur [58] to broad-spectrum, multi-site compounds and, more recently, to chemical agents with single-site activity. This specialization has created significant overlap between the modes of action of fungicides used in agriculture. Currently, more than half of global fungicide sales comprise sterol biosynthesis inhibitors (DMIs), such as azoles, and respiration inhibitors, including SDHIs and strobilurins. However, this reliance is increasingly challenged by several studies reporting steadily rising resistance to some of the most widely used fungicide classes and subclasses, including triazoles [14,59,60]. Because agricultural environments serve as selection grounds, understanding this emerging resistance, particularly among ubiquitous soil-borne pathogens such as *Fusarium* species, is critical for both crop protection and broader environmental and human health. A schematic representation of key antifungal resistance drivers in soil and potential effects on soil microbial communities is presented in Figure 2.

The development of resistance depends on several factors related to both the chemical agent (e.g., mode of action and efficacy) and the pathogen’s biology, including generation time, sporulation capacity, and epidemiology [61]. Although intensive chemical protection can double or even triple yields in crops such as rice and potatoes, most crops still reach only 60–75% of their maximum yield potential [62]. A major contributor to this gap is the persistence of resistant pathogen populations. This dynamic creates a feedback loop in which the degradation of arable land from intensive chemical use increases the need for intensified cultivation to sustain a growing population [63], further driving agrochemical demand and accelerating the evolution of resistance in phytopathogenic fungi. Consequently, the fungicide market, which Europe currently dominates, is projected to grow by over 7% each year until 2029 [64,65].

Despite this expanding arsenal of agrochemicals, fungal infections account for approximately 70% of major crop diseases [66], corresponding to global harvest losses of 20–40% (10–23% pre-harvest and 10–20% post-harvest) [66,67]. Among fungal pathogens, *Fusarium* species are of particular importance worldwide; the *F. graminearum* SC (FGSC) and the FOSC rank fourth and fifth, respectively, among the most significant fungal plant pathogens [1]. For example, some reviews report *Fusarium oxysporum* f. sp. *lycopersici* as responsible for about 14% of losses in tomato crops [68]. In addition to this pathogen, numerous other *Fusarium* species and formae speciales severely impact tomato production. These include *F. equiseti* and *F. oxysporum* f. sp. *radicis-lycopersici* (Forl), which cause Fusarium crown and root rot, *F. falciforme* and *F. solani* f. sp. *eumartii*, the causal agents of Fusarium foot rot; and *F. striatum*, which causes Fusarium crown and stem rot [68,69,70,71,72]. *Fusarium* species also pose a major post-harvest challenge due to fruit rot and the subsequent accumulation of mycotoxins in derived products; for example, an analysis of the Chinese market revealed that nearly 60% of all sampled tomato-based products were contaminated with *Fusarium* mycotoxins [73]. Similarly, cereal crops (particularly wheat, barley, and corn) are heavily impacted by FHB, primarily caused by *F. graminearum* [74], although the dominant causal species vary geographically. In Europe, for example, *F. graminearum*, *F. culmorum*, *F. poae*, and *F. avenaceum* are the most prevalent [75,76]. In addition to FHB, cereals are highly susceptible to other *Fusarium*-induced diseases, such as seedling blight, root rot, and Fusarium crown rot. As with tomatoes, these infections culminate in severe mycotoxin contamination, with trichothecenes such as deoxynivalenol posing the main challenge [77]. For example, *Fusarium* mycotoxins detected in Spanish cereal products have been shown to pose significant health risks to the general population [78]. In addition to public health concerns, contamination by *Fusarium* mycotoxins results in substantial economic losses, estimated at 3 billion euros in the European Union alone between 2010 and 2019 [79]. While chemical control can significantly reduce diseases such as FHB and lower mycotoxin production [80,81], its effectiveness is often limited. Historically, intensive chemical interventions, such as soil fumigation with methyl bromide to control *F. oxysporum* f. sp. *lycopersici* (Fol), have been employed; however, these methods are increasingly scrutinized due to environmental risks and the promotion of antifungal resistance [82,83,84]. Management is further complicated by the ecological versatility of *Fusarium* species, which are ubiquitous soil saprotrophs. Control strategies, such as crop rotation, often become ineffective in combating *Fusarium*-caused diseases, as many species can colonize alternative hosts or survive through spores that remain dormant in the soil for decades [85]. Moreover, even in crops where *Fusarium* is not the primary pathogen, the ubiquity of these fungi results in repeated exposure to fungicides used to control other diseases. This effect is exacerbated by long-term exposure to low fungicide doses due to accumulation in the soil matrix.

An additional factor influencing resistance dynamics is the degradation kinetics of fungicides in soil. Downstream effects on microbial diversity depend heavily on the persistence of the parent compound or its toxic degradation intermediates. Many fungicides are applied as commercial formulations designed for stability and solubility, which may contain co-formulants that can prolong the environmental persistence of the active ingredient and introduce their own toxicological effects on soil microbial diversity [86,87,88]. Over time, this contributes to ecotoxicological concerns associated with long soil half-lives. For instance, in Northern China, tebuconazole and difenoconazole have been detected at concentrations exceeding 0.1 mg kg^−1^ in over 1% of soil samples across multiple land-use types, demonstrating accumulation through repeated applications and slow degradation [89]. Importantly, toxicity is not limited to parent compounds. Although some degradation intermediates of difenoconazole are less toxic, others remain harmful to animal models [90]. The broader environmental fate of these subproducts and their specific effects on soil microbial communities remain poorly understood. A comprehensive understanding of exposure across the entire life cycle of fungicides is crucial to link environmentally relevant concentrations to the emergence and maintenance of antifungal resistance.

Beyond the direct selective pressure exerted by these chemicals, fungicide application indirectly influences pathogen dynamics by altering soil physiology, specifically by decreasing enzyme activity, respiration, and microbial carbon in a concentration-dependent manner. Impacts on microbial diversity vary with soil physicochemical characteristics, with studies reporting either smaller effects [91] or more pronounced disturbances [92] in soils with high organic matter and nitrogen content. Such compositional changes can, in turn, influence pathogen dynamics. The most commonly used fungicides are broad-spectrum, with largely unknown implications for the resistance of non-target species—either through direct exposure, non-target effects that confer adaptive advantages, or the disruption of soil microbial equilibrium [93,94]. Non-target effects have been observed in soils containing *Fusarium* and associated fungal communities [95]. Even pathogen-specific fungicides, such as phenamacril, have been linked not only to acquired resistance in *F. fujikuroi* but also to changes in soil microbial communities, suggesting compromised soil health [96]. Similarly, chlorothalonil and azoxystrobin negatively affect biological control agents of Fusarium wilt [97]. Beyond chemical inputs, agricultural management strategies fundamentally shape soil microbial communities. For instance, no-tillage systems appear to influence fungal communities less than conventional-tillage systems [98], possibly due to less physical disruption of the chitin cell wall, more stable temperature and moisture conditions [99] and less disruption of hyphal networks [100]. Disruption of soil microbial communities changes competition dynamics, potentially favoring resistant fungi [101,102]. While the influence of microbial community composition on *Fusarium* disease severity has been reviewed [103], a similar exercise focusing specifically on antifungal resistance dynamics remains lacking.

In addition to chemical inputs and micro-level soil management, the cultivation system itself represents an additional environmental factor that drives resistance. Greenhouse production systems are hotspots of antifungal resistance and may pose greater risks than open-field agriculture. High temperatures and humidity, intensive cultivation, and closed systems prevent leaching, promoting the accumulation of fungicides within confined soil volumes. Another issue is that crops grown in greenhouses are insulated from natural conditions and require intensive protection against opportunistic pathogens [104,105]. Conversely, disease management in enclosed systems presents distinct advantages that also influence resistance dynamics. Compared with open-field agriculture, pathogen control is often more precise, less dependent on weather conditions such as rainfall, and easier to deliver through irrigation-based applications [106,107]. Greenhouse production also frequently relies on soil replacement or soilless substrates [108], which can reduce *Fusarium* inoculum persistence and modify the selection pressures associated with resistance. Despite *Fusarium* being a prominent greenhouse pathogen [3], the risk and particularities of resistance in these systems remain underexplored.

### Insights into Antifungal Resistance from Clinical Studies

Crucially, the resistant pathogen populations cultivated in these agricultural hotspots do not always remain confined to the field. Clinical data provide insights into how agricultural soils serve as reservoirs of antifungal resistance. In the United States, clinical isolates of the FOSC and FSSC resistant to voriconazole and oxiconazole have been traced to regions with intensive agricultural azole use [109]. Similarly, in India, *F. falciforme* and *F. keratoplasticum* isolates obtained from keratitis patients exhibited high Minimum Inhibitory Concentration (MIC) values to triazoles and allylamines, consistent with the high MICs observed in environmental isolates from agricultural fields [110]. While the role of agricultural soils as reservoirs of resistant isolates of antifungal-resistant environmental fungi is well established for species such as *Aspergillus fumigatus* [111], similar environmental-to-clinical links remain largely unexplored for *Fusarium*. By contrast, clinical *Fusarium* isolates in this region have been studied primarily for their intrinsic resistance to many antifungals [1,2,3]. This disparity underscores the need for further studies examining *Fusarium* antifungal resistance within a One Health framework, particularly in European agricultural contexts.

## 3. Implications of Antifungal-Resistant *Fusarium* for Plant Disease Management

Managing *Fusarium* diseases remains a major challenge in plant pathology due to the genus’s genomic plasticity. While core housekeeping genes are stable, accessory chromosomes responsible for virulence and host specialization can rapidly evolve or transfer between strains, allowing the fungus to overcome the host immune system and develop fungicide resistance [112,113]. *Fusarium* species can persist in soil, plant residues, and seeds, while dissemination occurs through both sexual ascospores and asexual conidia, complicating single-target interventions [114,115].

### 3.1. Fusarium Disease Management Remains

#### 3.1.1. Chemical Control

Chemical fungicides have long been used to manage soilborne *Fusarium* diseases [116]. Early compounds, such as arsenic and methyl bromide, were effective but non-specific and environmentally hazardous [117,118]. Modern fungicides, including DMIs, MBCs, QoIs, and SDHIs, target discrete fungal pathways, offering improved efficacy and specificity [119,120,121].

Demethylation Inhibitors, the most widely used class of fungicides for chemical control of FHB in cereal crops [122], are often applied as foliar sprays, and their dominance is well-founded. In a quantitative review spanning 14 years of fungicide trials in Brazil, Machado et al. [123] concluded that a single spray of tebuconazole (triazole) at early wheat flowering reduced FHB by 59%, outperforming other fungicides, even when sprayed twice, such as carbendazim (benzimidazole), which achieved only a 55% reduction. The advantage became even more pronounced when assessing yield response. Two applications of tebuconazole, one at early flowering and a second 7–10 days later, provided the highest yield increase (19.2% over the control group), resulting in a gain of approximately 558 kg of wheat per hectare (ha), where carbendazim only saw an increase of 456 kg/ha.

However, it is important to note that fungicide efficacy varies several-fold among studies, even when using the same class, as it is highly dependent on environmental conditions and application timing [16,124,125]. Distinct efficacy disparities have been documented, with older triazoles (e.g., propiconazole) achieving only a 32% reduction in disease index, while newer compounds such as prothioconazole and metconazole registered inhibitions of up to 50% [16].

In FHB management, there is a narrow window to apply triazoles to effectively suppress the toxin DON, which is tied precisely to the flowering stage of the wheat, the most susceptible time for the crop to be infected by *F. graminearum* spores [124,125]. The fungicide needs to be present pre-emptively at sufficient concentrations to kill the fungus before it establishes itself in the plant’s vascular system, where control of FHB and suppression of DON falls several-fold [124].

Other groups of fungicides, such as MBCs, QoIs, and SDHIs, are mainly applied in rotation with DMIs to broaden the spectrum and mitigate resistance development [120,121]. While MBCs like benomyl were highly effective upon their introduction [126], the development of severe resistance and toxicological concerns has led to their restriction and decline in use [127]. The mechanisms and impact of MBC resistance are further discussed in Section 3.2.

Andrade et al. [50] tested over 200 *F. graminearum* strains that caused FHB in wheat and barley in Brazil against the two most used QoI fungicides in the market, azoxystrobin and pyraclostrobin. The study showed that the fungicide concentration required to inhibit growth or germination (EC_50_) was higher than in previous studies, such as those by Avozani et al. [128] and Duan et al. [48]. This finding could indicate that the systematic use of QoIs over the decades could be exerting a selective pressure resulting in more resistant *F. graminearum* strains [50].

In a recent study from China, seven *F. asiaticum* and six *F. graminearum* strains were tested for their sensitivity against five SDHIs (fluopyram, flutolanil, boscalid, benzovindiflupyr, and fluxapyroxad) [129]. The results highlighted a clear divergence in efficacy between more recent SDHIs (benzovindiflupyr) and older compounds. As in the case of the triazoles mentioned earlier, the newer fungicide formulations exhibited superior inhibition of spore germination and reduced DON contamination in *F. asiaticum*, while older compounds showed limited activity. Despite the success of newer SDHIs, like most fungicide classes mentioned so far in this review, SDHIs are single-site target fungicides and are classified as medium to high risk for developing resistance by the Fungicide Resistance Action Committee [130].

Single-target site fungicides truly changed the paradigm of plant disease management due to their specificity and effectiveness at controlling fungal phytopathogens [131]. Yet, they come at major risk of resistance development for the following reasons: (1) they exert tremendous selective pressure on the fungal population, killing almost all sensitive individuals; (2) spontaneous mutations in a single gene can often be enough to render the fungicide incompatible with the target site; and (3) pathogenic fungi tend to have plastic genomes characterized by a high content of transposable elements and, when exposed to sublethal doses of fungicides, may cause stress-induced genomic instability, which may accelerate the emergence of fungicide resistance [50,132,133]. Additionally, the widespread use of single-site target fungicides in crops can lead to cross-over into the clinical setting [134].

The strongest example of this occurs in azoles. Agricultural azoles (prothioconazole, tebuconazole) and clinical azoles (voriconazole, fluconazole) share the same CYP51 enzyme target. This is particularly worrisome for trans-kingdom fungal pathogens, which can infect plants and animals [135]. FSSC comprises many plant and opportunistic human pathogens, responsible for two-thirds of all fusarioses cases worldwide [136]. Although less common than invasive candidiasis and aspergillosis, human fusarioses can cause life-threatening opportunistic infections [137].

To mitigate the inherent problems with single-site target fungicides, farmers often resort to multi-site contact fungicides to preserve yield. They have the lowest intrinsic risk of resistance development. For a fungus to become resistant, it would require simultaneous mutations in several essential genes [138]. This event is genetically improbable and explains why chemical compounds such as mancozeb have remained effective for decades. In modern integrated pest management (IPM) strategies, mancozeb is routinely rotated with high-risk systemic fungicides, such as MBCs and Qols, to reduce selective pressures [139]. Despite its low propensity to develop resistance, the use of mancozeb is limited by its toxicity to animals and humans. The most serious health concerns are not with the chemical compound itself but with its primary breakdown product, ethylenethiourea, which is currently classified as an endocrine disruptor and potential carcinogen [140]. As such, the EU refused to renew the substance’s approval, and mancozeb has been effectively banned since January 2021, formalized in Commission Implementing Regulation (EU) 2020/2087 of 14 December 2020 [141].

Lastly, it is important to emphasize that plant disease management techniques are somewhat geography-specific, as they require analysis of soil, climate, plants, and the microbiome [142]. In the EU, the management of *Fusarium* diseases is supported by the common agricultural policy as part of the Farm to Fork Strategy, which emphasizes sustainable pesticide use, aims to minimize risks, and cuts chemical pesticide use by 50% by 2030 [143]. To meet these challenges, farmers and researchers are increasingly implementing IPM techniques tailored to control *Fusarium* and many other phytopathogens in agroecosystems [144]. Within this framework, chemical control remains an important tool when applied strategically. Fungicide rotations are essential to mitigate resistance development and should be implemented in combination with agronomic practices, resistant cultivars, and biological control methods to effectively control *Fusarium* diseases [145].

#### 3.1.2. Biological Control

Given the rapid emergence of fungicide resistance discussed in the previous section, the role of non-chemical strategies must be framed within the context of resistance management. These methods are thus foundational to mitigating antifungal resistance. Because biological agents and cultural practices rely on multi-modal mechanisms, such as spatial competition, antibiosis, and physical inoculum removal, they are resilient to the target-site mutations that typically affect chemical fungicides. Furthermore, by effectively reducing the overall *Fusarium* population size in the soil reservoir, these non-chemical strategies lower the probability of de novo resistance mutations arising. This way, they relieve the intense selection pressure placed on fungal populations, extending the operational lifespan of at-risk fungicide classes.

Biological control has emerged as a promising strategy for the integrated management of *Fusarium*, providing a sustainable alternative or complement to chemical fungicides [146]. This approach employs Biological Control Agents (BCAs), comprising antagonistic fungal and bacterial strains, to reduce inoculum levels and suppress infection through natural mechanisms such as competition, antibiosis, mycoparasitism, and activation of the host plant’s immune system [147]. Biological control aligns with IPM principles and provides the added advantage of enhancing plant and soil health, which, in turn, can increase crop yield [148]. For *Fusarium* spp., which often persist in soil and crop residues left on the field, BCAs are increasingly attractive because they can colonize the same niches as the phytopathogens, effectively suppressing re-infection [149].

Bacterial and fungal strains have been employed as BCAs to directly suppress *Fusarium* infections, including *Trichoderma* spp. [150], *Bacillus* spp. [151], *Streptomyces* spp. [152], *Pseudomonas* spp. [153], and even non-pathogenic strains of *Fusarium* [154].

Among the most prominent and well-documented BCAs is the genus *Trichoderma*, which already includes more than 300 molecularly characterized species. Their success as BCAs is largely attributed to their rapid mycelial development, high reproductive capacity, and ability to colonize multiple substrates [148]. This ecological flexibility is exhibited by *Trichoderma* spp. allows for rapid colonization of niches commonly exploited by *Fusarium*, restricting the phytopathogen’s access to space and nutrients needed for growth, and reducing inoculum persistence [155].

In vitro antagonism assays show *Trichoderma* spp. dominance over *F. oxysporum*. The rapid mycelial development of *Trichoderma reesei* effectively inhibited *F. oxysporum* growth by 62% after three days of incubation at 28 °C [156]. The study also demonstrated a synergistic effect between *T. reesei* and low doses of mancozeb (0.1 mg/mL). The fungicide had a negligible impact on the fungus, while enhancing its microparasitic capacity by 36% compared with tests in which *Trichoderma* was used alone. A similar scenario was seen when inoculating *T. asperellum* with *F. oxysporum* f. sp. *radicis-cucumerinum*, the competition between the species for nutrients and space resulted in the inhibition of colony radial growth of the pathogen by 85–89.2% [150]. This vulnerability to competition is particularly evident in tomato cultivation, where various biological alternatives (bacteria, fungi, plant and algal extracts) have demonstrated efficacy in controlling *Fusarium*-induced diseases, particularly in vitro and in greenhouse settings. The success of these interventions is partly due to the poor competitive fitness of certain tomato-infecting strains. For instance, both *F. oxysporum* f. sp. *radicis-lycopersici* (Forl) and *F. oxysporum* f. sp. *lycopersici* (Fol) have proven highly susceptible to competitive exclusion by microbial antagonists in experimental settings [157,158,159].

Starvation of essential nutrients, such as carbon, phosphorus, and iron, is the most common cause of death for microorganisms [160] and *Trichoderma* spp. show a superior capacity to mobilize and take up soil nutrients, derived from their ability to obtain ATP from the metabolism of multiple sugar sources, including chitin, the main component of fungal cell walls [160,161].

Iron competition plays a central role in inhibiting *Fusarium* spp. development in the soil. Some *Trichoderma* BCAs have the peculiarity of producing siderophores that chelate iron in the soil [162], a trait also observed in many endophytic bacterial BCAs, such as *Pseudomonas* spp. [153]. In a recent study, the inhibition mechanisms of *Pseudomonas* sp. P13 against *F. graminearum* were highlighted using multi-omics analysis. During its active logarithmic phase, P13 upregulates the biosynthesis of phenazine-1-carboxylate and hydrogen cyanide, potent antifungal metabolites that significantly inhibited *F. graminearum* growth. As the bacterial population reached the stationary phase, *Pseudomonas* sp. P13 downregulates toxin synthesis and upregulates the tricarboxylic acid cycle to fuel the secretion of high-affinity siderophores (e.g., histicorrugatin) that chelate environmental iron, creating an iron-deficient niche that effectively starves the fungal pathogen. This dual-phase adaptive strategy ultimately inhibited *F. graminearum* growth in vitro and in soil [153].

The mechanism of secretion of low-molecular-weight secondary metabolites, commonly referred to as antibiosis, is also observed in *Trichoderma* spp. [160,163] and *Bacillus* spp. [151]. These secondary metabolites are not directly involved in the organism’s development and are mostly synthesized as a stress response [164]. *Trichoderma* spp. are believed to biosynthesize approximately 1000 secondary metabolites [165], many of which exhibit antibacterial and antifungal activity. For example, 6-Pentil-2H-Pyran-2-one, produced by *T. atroviride* and *T. asperellum*, has proven highly effective against *F. oxysporum*, which is responsible for most cases of tomato wilt disease worldwide [166,167].

Fan et al. [151] demonstrated that the Fusarium wilt of banana can be effectively managed by two native *Bacillus* strains. *Bacillus amyloliquefaciens* YN0904 secreted a wide array of antimicrobial compounds associated with Non-Ribosomal Pathway Synthetases (NRPS) and Polyketide Synthases (PKS) pathways, which ultimately led to an inhibition of 82.6% in *F. oxysporum* f. sp. *cubense* in greenhouse experiments, while stimulating plant growth via auxin synthesis. The other strain was identified as *B. subtilis* YN1419 and exhibited a more specialized lifestyle, relying solely on the bacteriocin Subtisolin A (encoded by the *sboA* gene) to deform fungal hyphae, with a control effect of 85.6% against the pathogen.

A similar case is seen in some *Streptomyces* isolates. Kawicha et al. [152] performed in vivo assays under greenhouse conditions and confirmed that soil-derived *Streptomyces* isolates are effective BCAs against Fusarium wilt disease of tomato and banana. Their efficacy relies on the secretion of hydrolytic enzymes, mainly cellulase, to digest fungal cell walls while simultaneously releasing IAA, increasing the plant fitness.

This dual action, plant growth promotion and antibiosis, is characteristic of many endophytic Plant Growth Promoting Bacteria (PGPB); however, secondary metabolite production is highly strain- and environment-dependent [168]. Rather than being continuously expressed throughout the organism’s lifecycle, they are mainly secreted once the bacterial population has been established in response to specific environmental cues [164]. Thus, many endophytic PGPB and BCAs show their greatest efficacy when they colonize roots and internal tissues preventively or concomitantly with pathogen invasion [169]. Once *Fusarium* is systemically established in the vascular system, late BCA colonization rarely provides satisfactory control.

The studies mentioned so far demonstrate the potential of BCAs to suppress *Fusarium* diseases, but most results are from in vitro or greenhouse in planta assays, which do not reflect the complexity of field conditions. A major limitation in the current literature is the environmental context in which these evaluations are conducted. For example, despite promising results for biological control of *Fusarium* in tomatoes, approximately 79% of biocontrol efficacy trials have been conducted in controlled greenhouse conditions, with only 12% in open fields [170]. Consequently, translating in vitro and greenhouse efficacy into consistent field performance is the most significant challenge limiting large-scale BCA adoption, but other concerns regarding formulation stability, shelf life, and large-scale production further constrain widespread implementation [171]. Commercialization of BCAs remains complex and costly, largely due to regulatory requirements. However, a reasonable number of biologically based products have passed regulation and entered commercial use. Some commercialized fungal and bacterial BCAs used to control *Fusarium* spp. and other soilborne pathogens are listed in Table 2.

#### 3.1.3. Cultural Control 

In the context of resistance management, cultural control constitutes the first and most enduring line of defense against *Fusarium* diseases and represents a cornerstone of IPM strategies. By reducing baseline inoculum pressure, cultural practices minimize the frequency and dosage of fungicide applications required, thereby reducing the selective pressure that drives antifungal resistance. Unlike chemical or biological interventions, which act directly on the pathogen, cultural control involves agronomic and farming techniques that primarily aim to prevent disease establishment, reduce inoculum pressure, and simultaneously increase crop quantity and quality [172]. Preventive approaches are particularly relevant for soil- and residue-borne phytopathogens, which often persist for long periods. *Fusarium oxysporum* f. sp. *ciceri* chlamydospores were found to survive in the soil for more than six years [173]. In historically contaminated fields, eradication is rarely achievable; therefore, disease management strategies rely on lowering epidemic risk below economically damaging thresholds [144]. Within the EU, cultural control aligns closely with regulatory frameworks that prioritize prevention and sustainability, notably Directive 2009/128/EC, which promotes reduced pesticide dependence and favors agronomic solutions whenever possible [174]. Cultural practices are therefore not auxiliary measures, but rather the foundation upon which chemical and biological controls are built.

The deployment of resistant cultivars is widely regarded as the most effective and economically viable cultural measure for managing *Fusarium* diseases, particularly wilt and head blight in major crops [175]. The importance of using FHB-resistant wheat cultivars has been recognized for almost a century [176]. During the 1960s, intensive resistance breeding led to highly resistant cultivars, reducing disease prevalence and mitigating the use of chemical fungicides [144]. In *Fusarium* spp., host resistance reduces disease severity, limits pathogen colonization, and critically reduces mycotoxin accumulation in harvested products, safeguarding both yield and food safety [177]. However, resistance to *Fusarium* is rarely absolute. In most crops, it is quantitative and polygenic, involving multiple Quantitative Trait Loci (QTLs) that collectively moderate infection, symptom development, or toxin biosynthesis [178]. As has been reviewed, more than 400 QTLs have been identified using molecular mapping (e.g., [179]).

Historically, the development of FHB-resistant cultivars relied on conventional breeding, utilizing phenotypic selection across multiple environments to capture the quantitative nature of resistance [178]. Despite its time-consuming nature, this breeding method has produced wheat lines with the highest levels of FHB resistance, such as Sumai-3, which has been used as a parental line and a critical donor of FHB resistance in global breeding programs [180]. Nowadays, molecular marker-assisted breeding has become the central strategy in FHB-resistant programs. Unlike conventional breeding, this method enables breeders to track QTLs associated with FHB resistance independently of environmental variation [178]. The most prominent example is the *Fhb1* locus, originally identified in the Chinese wheat cultivar Sumai 3, which confers strong Type II resistance and reduces DON accumulation in the plant [179]. By utilizing genetic markers, Zhou et al. [181] reported that improved wheat lines carrying *Fhb1* (derived from the cultivar Ning 7840) exhibited a marked increase in FHB resistance, with the percentage of scabbed spikelets dropping to 30–40% from the 70–80% exhibited by the control group, lacking the resistance allele. Similar quantitative resistance frameworks apply to Fusarium wilt in tomato [182], banana [183], and legumes [175], where polygenic (or, in some cases, monogenic) resistance reduces vascular colonization, delays symptom onset, and limits systemic spread.

Despite their importance and efficacy, resistant cultivars alone are insufficient for long-term disease control. The high genetic diversity and adaptive capacity of *Fusarium* populations, coupled with environmental interactions, can compromise the durability of resistance. Consequently, resistant cultivars work best when integrated with complementary agronomic practices that actively reduce pathogen pressure and infection opportunities.

Crop rotation is an easy, cost-effective way to control diseases and is widely recommended as a cultural practice for managing FHB epidemics. Rotating susceptible crops with non-host or less suited hosts reduces the buildup of inoculum and disrupts pathogen life cycles [172,177]. The prevalence of *Fusarium* infections is directly correlated with the volume of crop residue left in the field post-harvest, with fungus-infected debris serving as a primary inoculum source [184]. Continuous monoculture of susceptible crops, such as wheat and maize, in both greenhouses and field systems creates genetically uniform host environments that foster the emergence and spread of resistant pathogen populations [185]. This practice significantly increases FHB pressure, often reaching thresholds that make yield loss inevitable. By growing non-host alternative crops (e.g., soybean), the epidemic cycle is disrupted, leading to drastic declines in pathogen populations within 2–3 years due to the absence of susceptible hosts [186].

Some guidelines suggest that any plant showing early symptoms of Fusarium wilt, such as yellowing of the leaves and stunted seedlings, should be excised and properly disposed of to prevent inoculum dispersal. Subsequently, a strict crop rotation regimen excluding the susceptible host species should be implemented for at least 4–5 years [172].

Sugar beets, flax, and alfalfa are considered clean-up or break crops that typically decrease FHB incidence in the field, as reviewed by Shah et al. [186]. However, their implementation does not come without risks. Tillmann et al. [187] reported that the incidence of *F. poae* and *F. tricinctum* increased in winter wheat grains following sugar beet. This suggests that the effectiveness of crop rotation depends on *Fusarium* spp. involved. While some species exhibit strong host-specificity, others, such as *F. oxysporum* and *F. solani*, possess broad host ranges and can persist saprotrophically in soil even in the absence of susceptible crops [114].

Soil tillage can also be an effective tool for managing *Fusarium* diseases, especially FHB in cereal crops. As established earlier in this review, crop debris serves as a primary inoculum source and is positively correlated with FHB prevalence and severity [184]. Conventional tillage directly addresses this problem by burying residues in the soil, accelerating decomposition, and mitigating the risk of aerial dispersion of chlamydospores, often resulting in lower disease pressure [188]. However, as discussed in Section 2, this practice involves an ecological trade-off, as conventional tillage severely disrupts soil microbial communities and hyphal networks [98,99,100], thereby altering competition dynamics and favoring the persistence of antifungal-resistant fungi in the soil reservoir [101,102].

Sowing date and fertilization also play a crucial role in the management of *Fusarium* diseases. In particular, high nitrogen fertilizer inputs have been linked to increased FHB and other foliar diseases (e.g., septoria tritici blotch) [189]. Field trials demonstrated that increasing nitrogen input from 70 to 170 kg N ha^−1^ increased *Fusarium* incidence across barley, wheat, and triticale [190].

Basic hygiene and sanitation-based practices can further reduce the spread of *Fusarium* infections at the field level. Farm equipment, footwear, and any tools utilized to come into direct contact with the soil should be adequately cleaned and disinfected, as they can inadvertently disseminate contaminated biomass between plots. Additionally, any diseased plant residues should be removed and properly disposed of or destroyed before decomposition begins, as incomplete decomposition may allow *Fusarium* to persist between seasons [172].

The type of irrigation system should also be considered carefully, as overhead irrigation may exacerbate disease pressure. It creates favorable conditions for both soilborne and foliar *Fusarium* infections, promoting leaf wetness and conidia splash dispersal [172]. Poorly drained or waterlogged fields can contribute to the development of root and crown rot [191]. To prevent this, drip or subsurface irrigation systems should be considered, as they reduce canopy humidity and conidia spread while improving water-use efficiency [172].

A pesticide-free weed control program should also be implemented to minimize pathogen survivability on the field [192]. Additionally, excessive handling of the crop should be avoided to minimize mechanical injuries. Common agricultural activities such as tying, thinning, and pruning can cause physical wounds if performed incorrectly, breaching the plant’s epidermal barriers and, in turn, facilitating the entry of Fusarium wilt pathogens. Special care must be taken to avoid root pruning during cultivation, as root cuts not only reduce plant vigor but also create openings for soilborne fungi [172].

Long-term *Fusarium* management requires integration across chemical, biological, and cultural methods, with cultural practices establishing a low-risk baseline, BCAs reinforcing ecological suppression, and fungicides applied strategically. Only through such multi-layered strategies can *Fusarium* diseases be effectively controlled while ensuring yield, food safety, and sustainability [112,114].

### 3.2. Impact of Antifungal Resistance in Fusarium on the Effectiveness of Disease Management

Despite the potential of cultural and biological control strategies discussed in Section 3.1, *Fusarium* disease management remains heavily dependent on fungicide applications. Chemical control remains a cornerstone of IPM programs, providing the most immediate and consistent reductions in disease incidence and severity, particularly under high pathogen pressure [193]. However, the increasing prevalence of fungicide-insensitive populations, together with the intrinsic multidrug resistance exhibited by many *Fusarium* spp., is progressively undermining the effectiveness and applicability of these compounds in the field [194].

Fungicide resistance is a stable, heritable genetic trait that reduces fungal sensitivity to a chemical compound, typically arising from mutations in genes encoding essential cellular targets [195]. From an evolutionary perspective, resistance does not enhance primary growth but rather confers a survival advantage under fungicide pressure [196]. Because resistance-associated mutations arise spontaneously [197], their emergence is difficult to predict, and once established at the field level, fungicides may shift from acting as lethal control agents to selective forces that favor resistant genotypes [198].

In *Fusarium*, resistance can be either intrinsic or acquired. Several species exhibited intrinsic resistance to DMIs [199], a fungicide class central to the management of FHB and other *Fusarium*-associated diseases [16,123]. In parallel, other species readily acquire resistance as a result of sustained, systematic fungicide use [50]. The occurrence, intensity, and stability of resistance vary widely across *Fusarium* spp., geographical locations, agronomic practices, and fungicide application regimes, making continuous resistance monitoring a critical component of effective disease management programs [200].

FHB remains one of the most economically devastating diseases in grain crops, with *F. graminearum* representing the primary pathogen of concern worldwide [50]. A wide range of fungicides, including DMIs [123], QoIs [50], and SDHIs [129], have been used to control this disease with moderate success in reducing visual symptoms and mycotoxin accumulation. Historically, MBCs were a major tool for this; upon their introduction in the 1960s, benomyl and its active metabolite, carbendazim, were one of the first truly effective, broad-spectrum systemic fungicides. However, due to their single-site mode of action, they develop resistance quickly in pathogenic fungi [194], which triggered one of the most severe cases of widespread fungicide resistance in agricultural history. This loss of efficacy, combined with subsequent toxicological concerns, led to its eventual restriction and decline in use [127]. For example, in China, MBCs, particularly carbendazim, were extensively used for four decades, but the widespread emergence of resistant members of the FGSC has severely constrained their utility, contributing to persistent FHB outbreaks in eastern China [201]. A study by Zhang et al. [201] indicates that multiple carbendazim-resistant genomes exist within the FGSC, suggesting that resistant populations have a higher fitness and could therefore be favored by natural selection. Despite the implementation of resistant cultivars and deep tillage practices, FHB incidence has increased in recent years, partly driven by climate change, resulting in yield losses approaching 20% across large production areas [201]. In the absence of effective biological control agents and with cultural practices failing to establish a sufficiently low infection baseline, growers increasingly rely on chemical control. This often leads to higher application rates, intensifying selection pressure and accelerating the spread of resistant populations [201].

At the molecular level, FGSC members harbor two homologous *β-tubulin* genes, *tub1* and *tub2*, with resistance to MBCs primarily associated with point mutations in *tub2* [202]. Field surveillance studies have consistently shown that amino acid substitutions at codons 167, 198, and 200 account for the vast majority of carbendazim-resistant genotypes [203,204]. Mutations such as F167Y and F200Y are commonly associated with moderate to high resistance levels [29,201,202], while substitutions at codon 198 can confer resistance phenotypes of varying magnitude depending on the specific amino acid change and genetic background [29,201].

The geographic distribution of resistant isolates strongly reflects historical fungicide use patterns. In Jiangsu Province, where carbendazim has been continuously applied for decades, MBC-resistant isolates are widespread, whereas no resistant genotypes were detected in regions without systematic carbendazim use [201]. Concerningly, all resistant *F. asiaticum* isolates identified in these surveys belonged to the 3-acetyldeoxynivalenol (3ADON) chemotype, suggesting that carbendazim applications may have actively selected for more aggressive and toxigenic populations. Although resistance mutations are frequently associated with fitness costs [205], this was not observed in these isolates, which instead exhibited enhanced growth and pathogenicity compared to wild-type strains [201]. Such traits may allow resistant populations to persist even in the absence of fungicide pressure, limiting the effectiveness of fungicide rotation strategies alone.

For wheat producers in China, the persistence of these resistant populations has already led to frequent control failures, with standard fungicide applications no longer delivering the expected levels of protection [203]. Genotypes carrying the F167Y mutation have become widely disseminated within carbendazim-resistant populations [202]. In practical terms, controlling such strains would require fungicide concentrations that are neither economically viable nor environmentally acceptable, eroding the cost-effectiveness of carbendazim and forcing producers to adopt more expensive multi-active formulations or abandon MBC-based fungicides altogether [206,207].

Beyond direct yield losses, fungicide resistance poses a serious threat to grain quality and marketability. Reduced fungicide sensitivity increases the likelihood of sublethal exposure, which in *F. graminearum* induces oxidative and osmotic stress responses that upregulate trichothecene biosynthesis genes [208,209]. Consequently, resistant populations may produce higher levels of DON and its acetylated derivatives, exacerbating food safety concerns [201]. Carbendazim-resistant strains have been shown to establish infections quickly and accumulate higher levels of DON in wheat grains than wild-type strains [206,210], underscoring the need for resistance-informed disease management strategies [200].

To compensate for the declining efficacy of older fungicides, newer compounds such as phenamacril have been introduced. Designed specifically to target *Fusarium* spp., phenamacril disrupts cytoskeletal function by inhibiting myosin-1 ATPase activity [211,212]. While effective at high application rates, resistance emerged rapidly following its introduction, driven by point mutations in the *myosin-5* gene [213]. Some mutations conferred high resistance without detectable fitness penalties, reinforcing the concept of the “pesticide treadmill”, where new fungicides provide only temporary relief before resistance emerges [214].

**Figure 3:**
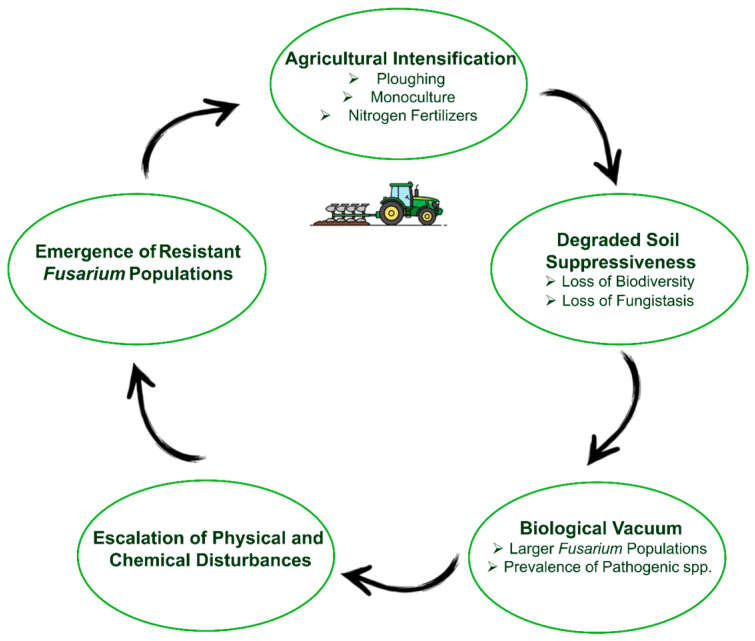
Conceptual representation of the degradation feedback loop linking agricultural intensification to the emergence of fungicide-resistant *Fusarium* populations in soil.

Collectively, these dynamics form a self-reinforcing degradation feedback loop in which agricultural intensification, escalating chemical inputs, and declining soil suppressiveness promote *Fusarium* dominance and accelerate the emergence of fungicide-resistant populations (Figure 3).

Overall, antifungal resistance in *Fusarium* fundamentally constrains disease management strategies. In high-pressure regions like eastern China, resistance is widely disseminated [202] despite the adoption of resistant cultivars and cultural control practices [201]. In the absence of effective biological alternatives, farmers remain locked into a cycle of escalating chemical inputs to sustain diminishing levels of control. Although the precise economic costs attributable solely to resistance are difficult to quantify, its practical consequences are evident: fungicides lose predictability as reliable safety nets and become context-dependent tools whose success hinges on local resistance profiles, environmental conditions, and application timing. These challenges underscore the urgent need for robust resistance-monitoring frameworks and for integrating non-chemical strategies that can reduce pathogen pressure and slow resistance evolution.

## 4. Innovative Plant Disease Management Approaches to Address Antifungal Resistance

New management strategies for crop protection are urgently needed to fill gaps left by current methods, particularly given the evolution of antifungal resistance. An increasingly popular strategy emphasizes sustainability, with IPM serving as the ultimate goal for deploying the most effective combination of measures. The promotion of IPM is a cornerstone of Directive 2009/128/EC, which defines the EU strategy for tackling plant pathogens, including *Fusarium*, in agroecosystems through a combination of agricultural practices, resistant cultivars, and both chemical and biological control methods [174]. IPM prioritizes non-chemical management and prevention, reflected in the promotion of cultural control methods. In this regard, sustainable plant disease management strategies have recently been reviewed elsewhere [200]. However, significant differences exist in how fungal pathogens and fungicide resistance are managed across EU members, influenced by climatic and social factors, leading to considerable variation in the implementation of integrated approaches [144].

A deeper understanding of sustainable practices (e.g., through meta-omics) is crucial for optimizing biocontrol and integrating it effectively into IPM. Biocontrol agents, such as *B. velezensis* SYL-3, employ both direct and indirect strategies to control *F. oxysporum*, thereby improving tobacco root development, enhancing soil physicochemical properties, and promoting beneficial changes to the rhizosphere microbial community [215]. Synergy has also been observed in nano-biocontrol combinations, such as nanoencapsulated Dimethachlon co-applied with *B. velezensis* MLY71 [216]. Meta-omics analysis has great potential for pathogen identification, the discovery of the molecular bases of disease, and the detection of microbial community changes that may signal plant vulnerability. For example, metatranscriptome analysis of an *F. oxysporum*-resistant bean cultivar revealed that resistance breeding co-selected for a rhizosphere microbiome enriched with beneficial bacteria expressing antifungal traits [217]. This knowledge is instrumental for plant microbiome engineering and the design of next-generation microbial inoculants [218,219].

Genomic approaches, including CRISPR/Cas9-based genome editing, are enhancing our understanding of the molecular mechanisms underlying *Fusarium* pathogenicity, facilitating the exploration of new disease control strategies and antifungal compounds [220,221]. Simultaneously, RNAi-based control strategies informed by insights into the evolution of fungicide resistance are being developed to silence essential genes. Examples include *AGO* and *DCL* genes in *F. graminearum* [222], *Faβ2Tub−3* in *Fusarium* spp. [223], and *FgCYP51* in *F. graminearum* [224]. RNAi targets also include toxin biosynthesis genes, such as *FUM1* or *FUM8* in *F. verticillioides* [225] and *TRI5* in *F. culmorum* [226]. However, resistance to RNAi-based agents can develop through point mutations [227], and field deployment faces challenges regarding specificity, durability, and dsRNA instability. Potential solutions include rotational applications of dsRNA sequences [228] and nanoparticle formulations [229], although regulatory frameworks are still being established. These innovative molecular strategies are increasingly vital for managing complex *Fusarium* infections in woody hosts, where traditional interventions like pruning or broad-spectrum fungicide applications are often limited or ineffective. For example, to control *F. circinatum* in pine, RNAi approaches such as Spray-Induced Gene Silencing (SIGS) are being developed to target and silence essential fungal genes [230], halting fungal growth without the environmental toxicity of traditional fungicides. For fruit tree pathogens like *F. mangiferae*, which can manipulate host growth by producing ethylene and disrupting host hormone balance, silencing the fungal genes involved in these processes presents a promising avenue for molecular-based, targeted control [231,232].

On the other hand, identifying alternative, eco-friendly biochemical strategies to manage *Fusarium* species has also become a priority. A variety of natural compounds, including plant extracts, essential oils, and microbial secondary metabolites, have demonstrated significant antifungal efficacy against plant-pathogenic *Fusarium* spp. Phenolic compounds such as thymol, eugenol, cinnamaldehyde, and carvacrol inhibit *F. graminearum* conidial germination and hyphal growth, with thymol and carvacrol consistently showing the strongest activity [233,234]. These compounds typically operate via multi-site mechanisms, including disruption of the fungal cell membrane and interference with ergosterol biosynthesis or ATP-related functions, leading to the leakage of intracellular components and subsequent cell death [235,236,237]. Because they do not rely on a single target site, the development of fungal resistance to these natural compounds is generally slower than that to single-site synthetic fungicides. In addition to botanical derivatives, microbial metabolites such as lipopeptides (e.g., iturin, surfactin, and fengycin) produced by antagonistic soil bacteria, such as *Bacillus* spp., represent a potent reservoir of natural antifungal agents capable of suppressing resistant *Fusarium* populations in the soil [238,239,240,241].

Functional peptides are increasingly valued as alternatives in plant disease management, offering diverse antimicrobial mechanisms against *Fusarium*. For example, surfactin A from *Bacillus* spp. show strong activity against *F. oxysporum*, *F. moniliforme*, and *F. solani* [242], Trichokonin VI from *T. pseudokoningii* induces cell death in *F. oxysporum* [243], and synthetic peptides like GV185 and GV187 demonstrate activity against *F. oxysporum* f. sp. *vasinfectum*, *F. verticillioides*, and *F. graminearum* [244]. While these peptides exhibit high activity and are environmentally safe [245], challenges persist regarding their large-scale production and degradation [246]. Research on nanoscale delivery systems of functional peptides has been developed as a possible solution to enhance stability [247]. Another avenue is drug repurposing, which involves screening existing compounds for antifungal activity to address the limited diversity of fungicides, thereby giving new life to often obsolete compounds. For instance, structural modification of the antibiotic kanamycin into the antifungal K20 showed in vitro inhibition of *F. graminearum*. In greenhouse trials, K20 reduced FHB severity, and in field trials, it acted synergistically with triazoles and strobilurin fungicides, reducing deoxynivalenol levels by 75% when combined with prothioconazole [248]. Similarly, synergists can reduce the doses of fungicides. *Taxodium* ‘zhongshansha’ essential oil showed synergistic potential with prothioconazole against *F. graminearum* in both in vitro and field trials in Anhui Province, China [249]. The mechanistic basis for such synergism often lies in targeted epigenetic or transcriptional modulation of fungal genes, an approach known as chemosensitization [250]. Natural plant-derived compounds such as phenols, flavonoids, and essential oils can effectively modulate *CYP51* expression and chemosensitize fungi to DMIs. Compounds like thymol appear to modulate the expression of ergosterol-biosynthesis genes, including *CYP51* and other *erg*-related genes, through transcriptional regulation rather than acting solely as direct CYP51 enzyme inhibitors, thereby interfering with the transcriptional responses the fungus uses to sense and compensate for ergosterol depletion [251,252]. By blocking these signaling pathways, these phytochemicals suppress the compensatory, stress-induced upregulation of *CYP51A* and reduce *CYP51B expression*, leading to impaired ergosterol biosynthesis, membrane dysfunction, and increased susceptibility to DMIs [236].

The application of nanotechnology in agriculture is emerging as a promising strategy to help circumvent traditional mechanisms of fungicide resistance in *Fusarium* populations. Nanoparticles (NPs) possess a high surface-area-to-volume ratio and unique physicochemical properties that enhance their interactions with fungal cells [253,254]. Metallic and metal-oxide nanoparticles, particularly silver (AgNPs), zinc oxide (ZnO), and copper-oxide (CuO) nanoparticles, exhibit strong antifungal activity against *F. graminearum* and other *Fusarium* species, particularly when combined with other existing management methods [255,256,257,258,259]. Their primary mechanisms of action include the induction of reactive oxygen species (ROS), oxidative damage to DNA and proteins, and disruption of cell-membrane integrity. Because these effects are multi-target and largely structural, they are less likely to be overcome by single-site resistance mutations, although stress-adaptation pathways can still evolve.

Although AgNPs show high activity against resistant *F. graminearum* strains, some studies report that they can induce or increase DON production under certain conditions, underscoring the need for optimization via combinations with conventional fungicides or careful dose–response tuning [256]. Polymeric nanomaterials such as chitosan nanoparticles are also being explored for their biocompatibility, as they can directly inhibit *Fusarium* growth and trigger defense-related responses in host plants, thereby supporting integrated disease-management strategies [99,260]. Precision management through the nanoencapsulation of existing active ingredients offers a promising avenue. For example, a recent nanoformulation of icosazole efficiently controlled *F. fujikuroi* spores while improving soil physicochemical and biological properties and exhibiting lower toxicity to zebrafish and rice seeds than the active ingredient alone. Notably, the nanoparticle coating degraded at lower pH levels, which favor the growth of *F. fujikuroi*, suggesting a potential for on-demand release [261]. However, industrial scalability and long-term effects require further testing. The biosynthesis of nanoparticles through bacterial, fungal, or plant systems offers a sustainable pathway. Examples include AgNPs synthesized by *Bacillus* sp. GP-23 [262] and *Aspergillus niger* [263], both of which show efficacy against *F. oxysporum*. Plant-mediated synthesis is a less-studied approach that involves producing copper oxide nanoparticles from, e.g., *Cassia fistula* leaf extract, thereby enhancing tomato defense mechanisms against *F. oxysporum* f. sp. *lycopersici* [264]. Agricultural waste, such as sugarcane bagasse, has also been utilized to manufacture silica nanoparticles that inhibit the growth of *F. oxysporum*, with beneficial effects on arugula growth and germination [265]. Despite these advances, concerns regarding bioaccumulation and toxicity persist, making the development of standardized guidelines for evaluating nanomaterials and their disposal a pressing need [266].

In situ, point-of-care diagnostic techniques are important because predictive approaches, although ideal, are not always possible. In this regard, techniques such as loop-mediated isothermal amplification (LAMP) enable quick screening. A LAMP assay utilizing diversity array technology and sequencing-derived DNA markers has been developed for the routine, unambiguous identification of *F. odoratissimum* (TR4) in banana plants under field conditions [267].

Finally, the integration of Artificial Intelligence (AI) and Machine Learning holds the potential to revolutionize crop management. AI and machine learning-powered systems can be utilized for the early detection of diseases. For instance, deep convolutional neural networks have been used to detect Fusarium wilt in banana from field images with high accuracy [268]. Furthermore, machine learning can help predict and assess the severity of *Fusarium* outbreaks. For example, several models were combined to predict the severity of chickpea wilt caused by *F. oxysporum* f. sp. *ciceris* [269], likely representing the first combined-model approach for predicting plant disease severity. AI also assists in optimizing biological parameters; for instance, AI-based modeling proved more efficient than statistical methods in optimizing the medium composition to enhance antifungal activity of *Streptomyces* sp. strain TN71 against *F. oxysporum* [270]. These technologies enable farmers to make timely, precise interventions, focusing on science-based approaches.

Despite the great potential of many of the approaches outlined, the literature indicates that some bottlenecks prevent their widespread adoption in agriculture today. A major limitation is the lack of standardized environmental safety data; for instance, the potential for off-target gene silencing by dsRNA, as well as the bioaccumulation and toxicity of metallic nanoparticles in soil ecosystems, remain poorly defined. It is thus crucial to establish unified regulatory guidelines tailored for nano-agrochemicals and RNAi agents. Research funding should be directed toward developing biodegradable nanocarriers that ensure the stable, targeted delivery of these active molecules without leaving toxic residues in the agroecosystem.

## 5. Detection and Monitoring of Antifungal Resistance in the Soil

Monitoring *Fusarium* populations for resistance is crucial to understanding, predicting, and counteracting the dynamics of pathogen development in the soil. Although many production systems design disease control strategies on a seasonal or annual basis, guided primarily by agronomic practices such as crop rotation, cultivar choice, and tillage [271,272], the emergence of AFR complicates these approaches. In several fungal pathogens, including *F. graminearum* and other cereal diseases, population sensitivity to commonly used fungicides can shift rapidly [14], underscoring the need for regular, field-level monitoring of fungicide sensitivity and resistance-associated markers. As a result, integrated disease management increasingly relies on fungicide rotation and the strategic use of products with different modes of action [273], which, in turn, demands continuous evaluation of efficacy, resistance risk, and application timing.

The traditional, locally oriented approach involves sampling representative areas within fields, isolating *Fusarium* strains, and subjecting them to phenotypic fungicide screenings [59,60]. However, soil is a highly heterogeneous matrix, leading to a patchy spatial distribution of the pathogen that complicates representative sampling, and isolating specific *Fusarium* strains from the complex soil microbiome requires specialized selective media and remains heavily time-consuming and labor-intensive. Consequently, sensitivity monitoring programs are not established in many regions, despite calls for action [139].

To overcome some of these limitations, efforts have been made to systematize the monitoring of *Fusarium*-caused diseases in crops by incorporating plant disease management strategies and climatic data. This has led to the development of predictive models, such as FusaProg [274]. While these models are useful for guiding the timing and dosage of fungicide applications, they are often limited by temporal and spatial variability [275,276]. In addition, they rarely incorporate real-time data on fungicide sensitivity, which limits their ability to predict failures driven by resistance.

To better address the limitations of phenotypic monitoring and predictive modeling, research has increasingly focused on molecular markers associated with resistance. As research on antifungal resistance is relatively recent, it has benefited substantially from a multi-omics approach, which is crucial for informing a more holistic understanding of resistance mechanisms and for detecting potential resistance biomarkers [277].

At the genomic level, biomarkers generally involve specific mutations in target genes. In *F. graminearum*, point mutations in *CYP51B*, such as Y137H, are critical markers of resistance to azoles like tebuconazole [278]. In the FSSC, reduced susceptibility to azoles is linked to point mutations in *CYP51A/B* and a specific 23 bp deletion in the *CYP51A* promoter region [279]. Transporter genes also play an important role, as exemplified by Ammar et al. [280].

At the transcriptomic level, resistance is often characterized by the overexpression of efflux pumps and stress response genes. Upregulation of *FgQdr2*, a plasma membrane drug/H+ antiporter, is associated with multi-drug resistance in *F. graminearum* [281]. Similarly, upregulation of *Tri12* in *F. oxysporum* and *F. proliferatum* has been linked to general resistance mechanisms [282]. It must be acknowledged, however, that applying transcriptomic approaches directly to soil environments remains a major research gap; *Fusarium* species often survive in soil as dormant chlamydospores [66], which limits the ability to capture active gene expression related to resistance in bulk soil prior to host infection.

Proteomic and metabolomic analyses further contribute to the identification of resistance-associated traits. At the proteomic level, biomarkers highlight stress responses, such as the high expression of metallopeptidase M35 in soil isolates of *F. oxysporum* under amphotericin B stress [283]. Metabolomic markers include the maintenance of ergosterol levels despite the presence of the drug, or the accumulation of precursors (eburicol) and toxic intermediates (14α-methylfecosterol) [19]. Furthermore, high levels of secondary metabolites can contribute to resistance by acting as competitive substrates or modulators of efflux pumps [284].

For routine detection of resistance-associated mutations, centralized, well-curated repositories are essential. Although most AFR mutations are thought to arise de novo rather than through horizontal gene transfer [285], current knowledge remains superficial and biased toward specific classes of antifungals. Current repositories are often outdated [286] or poorly annotated [287], hindering proper linkage of mutations to resistance outcomes. More recent initiatives, such as FungAMR, aim to annotate mutations based on the level of evidence supporting their roles in resistance [288]. It is therefore crucial that these databases remain adequately curated and inclusive of *Fusarium*-related mutations as they expand.

For fundamental discoveries linking genotype to phenotype, Genome-Wide Association Studies (GWAS) have been applied, for example, to FHB in Durum wheat [289]. Although GWAS approaches are powerful for identifying genetic determinants of resistance and pathogenicity [290,291,292,293,294,295,296], these large-scale experiments are expensive and are therefore better suited for mechanistic and exploratory research rather than routine monitoring.

Building on these advances, there is an increasing opportunity to develop robust spatiotemporal monitoring strategies. For example, integrating high-throughput genotyping with spore-capture technologies enables the association of fungicide-resistance alleles with disease levels and environmental conditions [297,298]. This approach is particularly promising for *Fusarium* species [299]. Transitioning these high-throughput molecular diagnostics directly to soil monitoring, however, presents distinct barriers. Extracting pathogen DNA directly from soil to detect single-nucleotide polymorphisms associated with resistance is complicated by the complex genomic background noise of the soil microbiome [300] and the presence of potent PCR inhibitors, such as humic acids [301]. Developing targeted extraction and amplification protocols that bypass these challenges remains a critical gap in AFR monitoring.

To translate molecular knowledge into practical disease management, it is essential to strike a balance between centralized, high-throughput efforts and rapid, decentralized diagnostic tools. Early detection is crucial for optimizing resistance management strategies [302]. Point-of-care diagnostic approaches, such as LAMP, enable decentralized genotyping and reduce reliance on expensive equipment and highly trained personnel, producing results through simple techniques, such as colorimetric or turbidity-based observations. For *Fusarium*, LAMP assays have been used to target *β*_2_-*tubulin* in *F. asiaticum* [303] and *F. graminearum* [203].

Emerging technologies are further expanding the boundaries of resistance monitoring. For instance, a rapid diagnostic tool for *Puccinia graminis* f. sp. *tritici* used a MinION nanopore sequencer to analyze a subset of highly polymorphic genes (*n* = 267) alongside azole- and SDHI-resistance targets [304]. This technique enabled rapid identification and was successfully tested in resource-limited areas of Ethiopia and Kenya. A similar pipeline could be adapted for *Fusarium* species, targeting resistance-associated genes based on more extensive omics efforts.

Ultimately, it is essential to acknowledge that monitoring programs cannot always adhere to a single, standardized framework. Instead, they must be adapted to specific objectives, such as evaluating management strategies, tracking evolutionary trends, or supporting rapid decision-making. As outlined by Zulak [305], this adaptation requires several key steps: (1) the development of accessible and standardized datasets on resistance mechanisms and spread, including harmonizing sampling methods and phenotypic assays; (2) the integration of highly technical, centralized approaches with regional and less technical efforts; and (3) the effective integration of genotype and phenotype data to inform management strategies.

With robust datasets, machine learning approaches can further boost the discovery of antifungal resistance biomarkers [306,307] and contribute to the systematization and optimization of phenotyping assays.

## 6. Conclusions

Antifungal resistance in soil strains of *Fusarium* arises from target-site mutations, multidrug efflux, genomic plasticity, and stress-adaptation networks, which are shaped by prolonged fungicide use and agricultural intensification. This makes soil a persistent reservoir for resistant lineages with pathogenic potential to major crops, directly linking the success of resistance management to food safety. At the same time, these persistent *Fusarium* pathogens may also pose risks to animals and humans.

Growing resistance is diminishing the reliability of major fungicide classes and driving a positive feedback loop of higher doses and costs, while other current interventions may not provide full or lasting control. It is therefore essential to invest in IPM strategies that respect the specificities of each region, combining emerging techniques with a deeper understanding and optimization of established practices to achieve meaningful improvement. In parallel, coordinated surveillance of antifungal resistance must be strengthened. Such surveillance should ideally combine field phenotyping with molecular markers and be supported by curated resistance databases, resource-efficient point-of-care detection tools, and well-tailored monitoring frameworks. We recommend the following key research and policy priorities: (i) clarifying the fitness costs and competitiveness of resistant *Fusarium* populations in natural soils; (ii) field-validating promising approaches (e.g., RNAi tools) and products (e.g., nano-based products), alongside robust assessment of environmental safety and scalability; and (iii) investing in tools that allow optimization of IPM components and democratization of resistance monitoring. Aligning these efforts with pesticide-reduction goals and sustainability policies will be critical. Ultimately, stronger coordination among the agricultural, environmental, and clinical sectors is urgently needed to break the cycle linking soil health decline, the spread of resistance, economic losses, and food safety risks.

## Figures and Tables

**Table 2:** Some commercialized fungal and bacterial BCAs, their target pathogens, and the mechanism of action.

Biocontrol Agent	Trade Name	Manufacturer	Target Pathogens/Diseases	Mechanism of Action
*Trichoderma virens* G41 + *T. harzianum* T-22	RootShield^®^ PLUS^+^	BioWorks Inc. (New York, NY, USA)	*Phytophthora*, *Pythium*, *Fusarium*, *Rhizoctonia*, *Cylindrocladium* and *Thielaviopsis* spp.	Mycoparasitism, competition, antibiosis, and induction of systemic resistance
*Trichoderma harzianum* T-22	TRIANUM^®^	Koppert Biological Systems (Berkel en Rodenrijs, The Netherlands)	*Fusarium*, *Pythium* and *Rhizoctonia* spp.	Mycoparasitism, competition, antibiosis, and induction of systemic resistance
*Trichoderma asperellum* T25 + *T. atroviride* T11	TUSAL^®^	TIMAC AGRO (Saint-Malo, France)	*Rhizoctonia solani*, *Sclerotinia sclerotiorum, Phytophthora, Fusarium* and *Pythium* spp.,	Mycoparasitism, competition, antibiosis, and induction of systemic resistance
*Trichoderma asperellum* TV1	PATRIOT GOLD^®^	Sumitomo Chemical Agro Europe (Zaventem, Belgium)	Soilborne fungal diseases (*Fusarium*, *Pythium* and *Verticillium* spp.)	Mycoparasitism, competition, antibiosis, and induction of systemic resistance
*Trichoderma asperellum ICC 012* + *T. gamsii ICC 080*	REMEDIER^®^	Gowan Crop Protection Ltd. (Harpenden, UK)	Wilt and root diseases (*Rhizoctonia solani*, *Fusarium* spp. and other soilborne pathogens)	Mycoparasitism, competition, antibiosis, and induction of systemic resistance
*Clonostachys rosea* J1446	LALSTOP G46^®^	Lallemand Plant Care (Danstar Ferment AG, Zug, Switzerland)	Wilt and root diseases (*Rhizoctonia* spp., *Fusarium* spp., *Botrytis cinerea*, *Didymella bryoniae*)	Mycoparasitism, competition and antibiosis
*Trichoderma asperellum* T34	T34 BIOCONTROL^®^	Biocontrol Technologies S.L. (Barcelona, Spain)	Wilt and root diseases *(Fusarium* and, *Pythium* spp.)	Mycoparasitism, competition, antibiosis, and induction of systemic resistance
*Pseudomonas chlororaphis* MA342	Cedomon^®^/ Cerall^®^	Koppert Biological Systems (Berkel en Rodenrijs, The Netherlands)	Seedborne and soilborne *Fusarium* and other fungi	Competition, antibiosis and induction of systemic resistance
*Bacillus amyloliquefaciens* QST 713	SERENADE ASO^®^	Bayer CropScience (Monheim am Rhein, Germany)	Large spectrum activity	Competition, antibiosis, and induction of systemic resistance
*Pseudomonas* sp. DSMZ 13134	PRORADIX^®^	Sourcon Padena GmbH (Tübingen, Germany)	*Helminthosporium solani*, *Fusarium* and *Rhizoctonia* spp.	Competition and antibiosis
*Streptomyces* sp. K61	LALSTOP K61^®^	Lallemand Plant Care (Danstar Ferment AG, Zug, Switzerland)	Wilt and root diseases (*Alternaria*, *Phytium*, *Fusarium* and *Rhizoctonia* spp.)	Mycoparasitism, competition and antibiosis
*Streptomyces lydicus* WYEC 108	ACTINOVATE^®^	Novonesis (Bagsværd, Denmark)	Root diseases and airborne pathogens (*Fusarium*, *Botrytis* and *Alternaria* spp.)	Competition, antibiosis, and induction of systemic resistance

## Data Availability

No new data were created or analyzed in this study. Data sharing is not applicable to this article.
